# Freeze-Dried Powder of *Rubus coreanus* Miquel Ameliorates Isoproterenol-Induced Oxidative Stress and Tissue Damage in Rats

**DOI:** 10.4014/jmb.2106.06047

**Published:** 2021-06-30

**Authors:** Jin Tae Kim, Shuai Qiu, Yimeng Zhou, Ji Hyun Moon, Seung Beom Lee, Ho Jin Park, Hong Jin Lee

**Affiliations:** Department of Food Science and Biotechnology, Chung-Ang University, Anseong 17546, Republic of Korea

**Keywords:** Bokbunja, isoproterenol, myocardial infarction, oxidative stress, *Rubus coreanus* Miquel

## Abstract

*Rubus coreanus* Miquel (bokbunja), Korean black raspberry, is known to possess various phytochemicals that exert antioxidative, anti-inflammatory, and anti-cancer effects. However, most studies on *Rubus coreanus* Miquel have been performed with the solvent extracts and/or a single component to demonstrate the efficacy, while studies evaluating the effect of the whole fructus of *Rubus coreanus* Miquel are limited. In this study, therefore, we employed the isoproterenol (IPN)-induced myocardial infarction model and investigated the effect of freeze-dried powder of *Rubus coreanus* Miquel (RCP) on oxidative stress and prevention of organ damage. Oral administration of RCP reduced the level of toxicity markers, alanine transaminase (ALT), aspartate transaminase (AST), and lactate dehydrogenase (LDH) without affecting body weight and diet intake. The oxidative stress marker glutathione (GSH) increased about 45% and malonaldehyde (MDA) decreased about 27% compared to the IPN group with RCP-H (3%) administration. By histological analysis, IPN induced significant myocardial damage in the heart and vascular injury in the liver, and RCP administration ameliorated the damages in a dose-dependent manner. Taken together, RCP activated the antioxidant system leading to prevention of damage to organs by IPN in rats, making it possible to expect beneficial efficacies by consuming the whole fructus of *Rubus coreanus* Miquel.

## Introduction

*Rubus coreanus* Miquel (bokbunja), Korean black raspberry, is native to Northeast Asian countries including Korea and has been used in therapeutics in Asian traditional medicine, according to ‘Dongui Bogam,’ a Korean traditional book of medicine. Many studies have recently demonstrated that the different extracts of *R coreanus* Miquel possess health-benefiting efficacies and can ameliorate oxidative stress, inflammation, liver steatosis, obesity, osteoporosis, and cancer [1-7]. For example, the methanol extract prevented osteoporosis via balanced osteoblasts and osteoclasts in in vitro and in vivo models [5] and exerted a strong anti-inflammatory effect via suppressing the expression of pro-inflammatory mediators and activating hemoxygenase-1 signaling [1, 2]. In addition, the ethanol extract also inhibited adipogenesis in 3T3-L1 cell lines and suppressed fat accumulation in high-fat diet-induced animal model via increased thermogenesis activity [6]. Interestingly, the water extract of *Rubus coreanus* Miquel was one of the top rankers in inhibiting three different oxidation reactions among seventy Korean traditional herbs [8]. However, most of these studies were performed with the different extracts whereas the study investigating the effect of the whole fructus of *Rubus coreanus* Miquel has been limited.

To determine the antioxidative effect of the whole fructus of *Rubus coreanus* Miquel, we prepared a freeze-dried powder (RCP) and employed an animal model where the oxidative stress is induced by isoproterenol (IPN). IPN, a β-adrenoceptor (β-AR) agonist, is known to induce myocardial infarction through oxidative stress in the cardio system of rats [9]. β-AR stimulation contributes to β-AR-mediated oxidative stress, leading to a disturbance in the physiological balance between the production of free radicals and an antioxidative defense system [10]. The endogenous antioxidant marker glutathione (GSH) and enzymes such as superoxide dismutase (SOD), catalase (CAT), glutathione-S-transferase (GST) were also modulated by heart hypertrophy [11]. In addition, lipid metabolism was reported to be regulated by lipid peroxidation [12]. Lipid peroxidation activated under oxidative stress conditions causes cardiovascular damage and is known to interfere with normal lipid metabolism [13]. There are several studies that have used the IPN-induced oxidative animal model to determine the antioxidant activities of natural polyphenolic antioxidants [14]. For example, the oral administration of ellagic acid prevented myocardial damage by restoring the expression levels of antioxidant enzymes as well as their biological activities and reducing the levels of lipid peroxidation [15]. However, the effect of RCP on IPN-induced oxidative stress and tissue damage in rats has not been studied.

In this study, we evaluated the effect of RCP on prevention of heart and liver damage induced by IPN treatment in SD rats, and confirmed the regulatory activities on the anti-oxidative system and/or lipid metabolism.

## Materials and Methods

### RCP and Diet Preparation

*Rubus coreanus* Miquel was purchased from the local market at Gochang (Korea), and freeze-dried for 100 h under pressure lower than 0.005 mBar (IlsinBioBase, Korea). After pulverization, the powder was stored at -20°C. Then, RCP was mixed with chow diet containing crude protein (18.6%), fat (6.2%) and carbohydrate (44.2%)(Envigo, USA), to prepare the diets of RCP-L (0.3%), RCP-M (1.0%), and RCP-H (3.0%) (DooYeol Biotech, Korea).

### Animal Experiment

Adult male Sprague-Dawley (SD) rats (150–200 g) were purchased from DooYeol Biotech, and randomly divided into five groups; Con (*n* =4), IPN (*n* =7), IPN+RCP-L (*n* =6), IPN+RCP-M (*n* =7), and IPN+RCP-H (*n* = 7). They were housed under controlled conditions of relative humidity (50 ± 10%), and room temperature (25± 5°C) with 12 h light and dark cycles. After one week of adaptation, the rats were fed with the assigned diet, and body weight and diet intake were measured every week. All animal experiments were performed according to guidelines approved by the Animal Care and Use Committee at Chung-Ang University (Approval No. 2020-00106).

### Induction of Oxidative Stress and Tissue Damage

Isoproterenol (IPN) purchased from Sigma-Aldrich (USA) was prepared by dissolving in phosphate-buffered saline (PBS). Two days before sacrifice, the rats were subcutaneously injected twice with IPN (65 mg/kg BW) at an interval of 24 h for the induction of oxidative stress and myocardial infarction. All animals were then sacrificed and blood samples were taken for serum analysis and the hearts and livers were collected for histological analysis. Each organ was weighed and stored in 10% formalin (Sigma-Aldrich).

### Hematoxylin & Eosin (H&E) Staining

The heart and liver tissues in 10% formalin were embedded in paraffin block after the dehydration process and cut into 5-μm-thick tissue sections using a microtome (Leica, Leica Biosystems, Germany). The tissue sections were rehydrated and stained with hematoxylin for nuclear staining and eosin for the cytoplasm for counter staining. The tissues were then examined under a light microscope (Olympus, Japan) for histological changes and pictures were taken.

### Serum Analysis

After blood collection, serum was taken by centrifugation at 2,000 × *g* for 10 min at room temperature. Serum was analyzed using a biochemical blood analyzer (BS220, China). The serum levels of different markers were measured: alanine transaminase (ALT), aspartate transaminase (AST), lactate dehydrogenase (LDH), triglyceride (TG), total-cholesterol (T-Chol), low-density lipoprotein (LDL), and high-density lipoprotein (HDL).

### Enzyme-Linked Immunosorbent Assay (ELISA)

To evaluate the oxidative stress markers in serum, ELISA was performed. Following the manufacturer’s guidance, the serum was incubated in wells pre-coated with antibody and treated with primary and horseradish peroxidase (HRP)-conjugated secondary antibody. Then, substrate solution was added and the developed color was measured at 450 nm (Molecular Devices, USA). ELISA micro-plate assay kits for detecting GSH (Abbexa Ltd., UK) and MDA (MyBioSource, USA) were used.

### Statistical Analysis

The statistical differences were analyzed by IBM SPSS Statistics v.20 software (USA), and mean differences were analyzed using one-way analysis of variance (ANOVA) with Duncan’s post hoc test. Significant difference was at *p* < 0.05.

## Results and Discussion

### RCP Reduced the Heart Weight Increased by IPN Injection without Affecting the Body Weight in SD Rats

Subcutaneous injection of IPN in rats was reported to increase heart rate and increase the heart weight, where IPN produced greater hypotension, followed by induction of reflex compensatory responses such as cardiac failure [16]. An increase in heart weight is attributed to increase of water content, edematous intramuscular space, and protein content in the heart tissue [17], where 1% increase of myocardial water content resulted in a possible 10% reduction in myocardial function [18]. Also observed were extensive edematous intramuscular space, accumulation of mucopolysaccharides, and cellular infiltration after the induction of myocardial infarction [19].

**Fig. 1:**
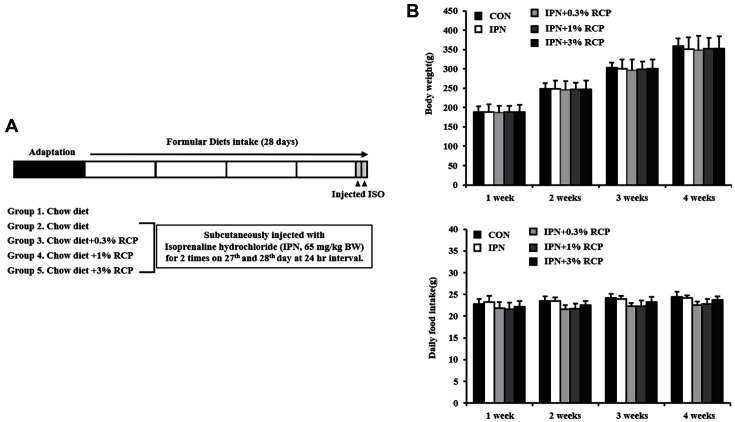
Scheme of animal experiment and the changes of body weight and daily food intake. (**A**) Scheme of the animal experiment. (**B**) Changes of average body weight and food intake during the experiment. Values represent mean ± SD. Different lowercase letters (a-c) indicate statistically significant differences at *p* < 0.05 evaluated by one-way ANOVA followed by Duncan test.

Here, we confirmed that IPN significantly increased the heart weight as well as the relative heart weight (Table 1). However, the body weight and liver weight were not affected by IPN injection and RCP administration (Fig. 1B and Table 1). The food intakes between groups were not significantly different (Fig. 1B), as indicated by the average consumption of RCP at 70 ± 3, 220 ± 14, and 69 ± 31 mg/day in RCP-L (0.3%), RCP-M (1.0%), and RCP-H (3.0%) groups, respectively. As shown in Table 1, RCP administration significantly suppressed the heart enlargement in a dose-dependent manner from 1.56 ± 0.15 g in IPN group to 1.40 ± 0.06 g in RCPH group, as well as the relative heart weight from 0.43 ± 0.06 g/100 g BW to 0.38 ± 0.02 g/100 g BW, respectively. These results suggest that RCP activates a defense system in vivo, leading to counteract the heart damage by IPN.

### RCP Ameliorated the Liver Toxicity Derived from IPN-Induced Myocardial Injury in SD Rats

IPN is proposed to act as a cardiotoxic agent due to its destruction of myocardial cells, where the severity of necrotic damage in the myocardial membrane was observed [20]. As shown in Table 2, IPN treatment significantly increased the levels of well-known toxicity markers, ALT, from 42.25 ± 6.67 U/L to 108.21 ± 37.10 U/L, AST, from 127.28 ± 38.86 U/L to 781.93 ± 430.56 U/L, and LDH, from 1,132.75 ± 325.89 U/L to 2140 ± 551.59 U/L in serum, which is in line with the previous study [20]. Oral administration of RCPs significantly reduced levels of AST, ALT, and LDH in a dose-dependent manner (Table 2). Especially in RCP-H group, the level of AST, ALT, and LDH was decreased to 42.22%, 30.23%, and 41.97%, respectively, compared to IPN group (Table 2). Interestingly, the level of ALT in RCP-H group was reduced to the level of control group (Table 2). There are many studies demonstrating the effects of berry extracts and their constituents on ameliorating the toxicity in different in vitro and in vivo models, mainly by reducing the oxidative stress [21-23]. The current study also suggests the protective effect of RCP in IPN-induced cardiac injury may derive from the antioxidative activity.

### RCP Reduced the Oxidative Stress Derived from IPN-Induced Myocardial Injury in SD Rats

In IPN-induced cardiac injury model, IPN was reported to trigger the production of reactive oxygen species (ROS) by depleting the endogenous antioxidant system [24]. GSH is one of the phase II detoxifying enzymes, and MDA is produced from lipid peroxidation of polyunsaturated fatty acids, indicating that GSH and MDA are well-known indicators of oxidative stress [25]. Here, we found that IPN significantly increased the level of MDA and decreased the level of GSH in the serum of rats which is consistent with previous studies [26, 27]. As shown in Table 3, oral administration of RCP counteracted the IPN regulation. Especially in RCP-H group, the level of GSH reduced by IPN was recovered from 4.17 ± 0.48 μg/ml to 6.03 ± 0.79 μg/ml, and that of MDA increased by IPN was reduced from 2.46 ± 0.45 nmol/ml to 1.80 ± 0.15 nmol/ml in the serum (Table 3).

It has been demonstrated that β-adrenoreceptor stimulation by IPN provokes cardiac oxidative stress [28]. The elevated levels of lipid peroxidation by ROS may decrease mitochondrial membrane fluidity, increase the negative surface charge distribution, and alter membrane ionic permeability, including proton permeability, which uncouples oxidative phosphorylation [29], and different polyphenols suppressed the production of IPN-induced lipid peroxides [30, 31]. Therefore, our data suggest that RCP activates the antioxidant system and minimizes the production of ROS caused by IPN in rats.

### RCP Improved the Blood Lipid Profiling Derived from IPN-Induced Myocardial Injury in SD Rats

Abnormalities in lipid metabolism induced by isoproterenol were observed to cause a rise in the serum levels of phospholipids, lipid peroxides, LDL, and VLDL as well as a decrease in the serum level of HDL, which was paralleled by abnormal activities of lipid metabolizing enzymes [32]. In the IPN-injected rat model, it was reported that the levels of TG, LDL and VLDL were significantly increased whereas that of HDL was decreased compared to the control [33]. As shown in Table 3, IPN significantly decreased the serum level of HDL known to be involved in the transport of cholesterol from tissues to the liver for its catabolism [34], and RCP exerted significant recovery, almost to the level of the control, in a dose-dependent manner. Especially, HDL level in IPN group and RCP-H group was 22.71 ± 5.88 mg/dL and 33.87 ± 5.63 mg/dL, respectively (Table 3). In case of TG and LDL, IPN significantly increased their serum level. However, the level of TG and LDL in RCP-administered group was not significantly different from those in IPN group although their average values were lower than those in IPN group.

### RCP Improved the Liver and Heart Tissue Damage Derived from IPN-Induced Myocardial Injury in SD Rats

**Fig. 2:**
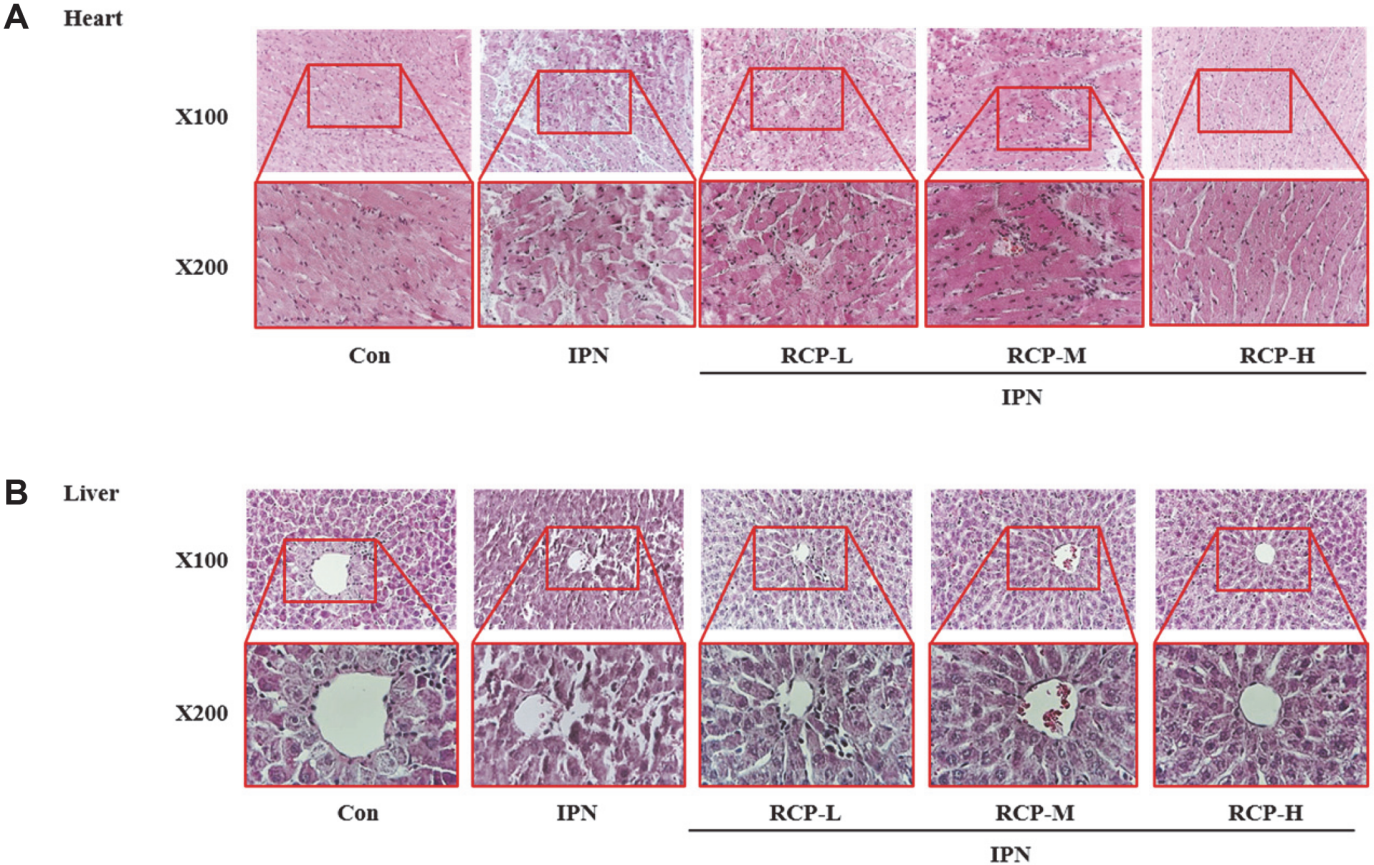
The protective effect of RCP on tissue damage induced by IPN in rats. The heart (**A**) and the liver tissue (**B**) were stained by H&E staining as described in Materials and Methods, and a representative picture was taken under microscope (magnification, 200×).

Oxidative stress induced by IPN can cause irreversible damage in the heart while promoting necrosis and increasing fibrosis in both heart and liver tissues [35]. The produced oxidated quinones react with oxygen and ROS such as O_2_^-^ and H_2_O_2_ and cause myocardial infarction leading to the development of infarct-like necrosis [36]. Here, we evaluated the tissue quality in the liver and heart by H&E staining. As shown in Fig. 2, the treatment of IPN induced significant myocardial architecture damage including inflammation in the heart and vascular injury in the liver. However, the histologic injuries were ameliorated by the RCP administration in a dose-dependent manner (Fig. 2). Especially, RCP-H exerted significant protection from IPN-induced organ damages to similar levels compared to the control.

Various polyphenols such as fisetin and EGCG were reported to restore inotropy and reduce apoptosis and necrosis under IPN-induced oxidative stress, demonstrating the involvement of antioxidant markers GSH and GPx [37, 38]. The condensed juices of *R. coreanus* Miquel effectively prevented oxidative stress damage to liver tissue induced by carbon tetrachloride (CCl_4_) [21], and its seed oil inhibited ROS-induced oxidative damage in the liver cells by enhancing cellular antioxidant enzyme activity [39]. In line with the previous studies, our results also suggest that RCP protects against organ damages via activating the antioxidant system.

*R. coreanus* Miquel was reported to contain higher amounts of polyphenols, flavonoids and anthocyanins compared to other berries and exert the strongest antioxidant activity among seventy Korean traditional herbs [8]. Among the different phytochemicals, ellagic acid was identified as one of the key constituents in *R. coreanus* Miquel, corresponding to about 408.57 mg/kg fresh weight [40], suggesting ellagic acid as a potent candidate for antioxidant activity. Interestingly, oral administration of ellagic acid (7.5 and 15 mg/kg) daily for a period of 10 days significantly recovered electrocardiogram pattern, arterial pressure, and heart rate in IPN-induced myocardial infarction in rats while also regulating the levels of lipid peroxidation and antioxidant markers in the damaged heart tissue [15]. Therefore, it is possible that ellagic acid is the main constituent in RCP exerting the activities to prevent heart and liver damage and reduce oxidative stress.

In conclusion, freeze-dried powder of *R. coreanus* Miquel exerted significant effect to ameliorate IPN-induced tissue damage in the heart and liver while also regulating antioxidant activity and lipid metabolism. It is well known that berries including *R. coreanus* Miquel possess antioxidant activity. However, to our best knowledge, ours is the first report to investigate the effect of the whole fructus in IPN-induced myocardial infarction animal model. Although more detailed mechanistic studies are necessary, the findings presented here provide significant evidence for the health-benefitting efficacy of *R. coreanus* Miquel by whole fruit consumption.

## Figures and Tables

**Table 1:** The effect of RCP on body and organ weights of rats in IPN-induced myocardial injury model.

	Control	IPN	IPN + RCP-L	IPN + RCP-M	IPN + RCP-H
Body weight (g)	385.06±3.55	369.55±26.88	366.44±42.11	366.67±23.49	373.98±29.53
Heart weight (g)	1.16±0.06^c^	1.56±0.15^a^	1.44±0.12^ab^	1.43±0.08^ab^	1.40±0.06^b^
Liver weight (g)	12.86±0.36	12.76±1.28	13.86±1.60	13.07±1.80	13.97±1.40
Relative heart weight (g/100 g)	0.30±0.01^c^	0.43±0.06^a^	0.40±0.03^ab^	0.39±0.02^ab^	0.38±0.02^b^
Relative Liver weight (g/100 g)	3.34±0.10	3.48±0.49	3.85±0.74	3.56±0.41	3.73±0.18

The results are expressed as mean ± SD in each group.

Values not sharing a common superscript (a, b, c) differ significantly with each other at *p* < 0.05.

**Table 2:** The effect of RCP on serum markers of liver toxicity in IPN-induced myocardial injury model.

	Control	IPN	IPN + RCP-L	IPN + RCP-M	IPN + RCP-H
ALT (U/L)	42.25±6.67^c^	108.21±37.10^a^	74.87±38.39^ab^	72.14±41.50^ab^	45.69±12.39^bc^
AST (U/L)	127.28±38.86^b^	781.93±430.56^a^	389.58±139.34^ab^	372.33±253.85^ab^	236.84±68.88^ab^
LDH (U/L)	1132.75±325.89^b^	2140.00±551.59^a^	1627.67±350.80^b^	1572.29±551.62^b^	897.86±358.37^b^

ALT: alanine transaminase, AST: aspartate transaminase, LDH: lactate dehydrogenase.

The results are expressed as mean ± SD in each group.

Values not sharing a common superscript (a, b, c) differ significantly with each other at *p* < 0.05.

**Table 3:** The effect of RCP on serum markers of oxidative stress and lipid metabolism in IPN-induced myocardial injury model.

	Control	IPN	IPN + RCP-L	IPN + RCP-M	IPN + RCP-H
Glutathione (ug/mL)	5.68±0.20^a^	4.17±0.48^b^	5.52±0.83^a^	5.75±0.66^a^	6.03±0.79^a^
Malondialdehyde (nmol/mL)	1.60±0.13^b^	2.46±0.45^a^	2.08±0.48^ab^	2.08±0.37^ab^	1.80±0.15^b^
T-Chol (mg/dL)	57.45±3.03	50.18±9.09	56.97±6.58	55.94±9.29	60.86±7.36
TG (mg/dL)	38.51±12.20^b^	139.14±36.33^a^	154.63±18.72^a^	107.40±55.30^a^	129.86±40.91^a^
LDL (mg/dL)	13.46±1.89^b^	21.53±7.86^a^	20.56±4.76^a^	17.96±2.68^ab^	19.74±2.14^ab^
HDL (mg/dL)	39.42±0.71^a^	22.71±5.88^c^	27.80±2.05^bc^	30.50±6.10^bc^	33.87±5.63^ab^

TG: triglyceride, T-Chol: total-cholesterol, LDL: low-density lipoprotein and HDL: high-density lipoprotein.

The results are expressed as mean ± SD in each group.

Values not sharing a common superscript (a, b, c) differ significantly with each other at *p* <0.05.

## References

[1] Park JH, Oh SM, Lim SS, Lee YS, Shin HK, Oh YS (2006). Induction of heme oxygenase-1 mediates the anti-inflammatory effects of the ethanol extract of *Rubus coreanus* in murine macrophages. Biochem. Biophys. Res. Commun..

[2] Lee JE, Cho SM, Park E, Lee SM, Kim Y, Auh JH (2014). Anti-inflammatory effects of *Rubus coreanus* miquel through inhibition of NF-kappaB and MAP Kinase. Nutr. Res. Pract..

[3] Bhandary B, Lee GH, Marahatta A, Lee HY, Kim SY, So BO (2012). Water extracts of immature *Rubus coreanus* regulate lipid metabolism in liver cells. Biol. Pharm. Bull..

[4] Lee KH, Jeong ES, Jang G, Na JR, Park S, Kang WS (2020). Unripe *Rubus coreanus* miquel extract containing ellagic acid regulates AMPK, SREBP-2, HMGCR, and INSIG-1 signaling and cholesterol metabolism in vitro and in vivo. Nutrients.

[5] Do SH, Lee JW, Jeong WI, Chung JY, Park SJ, Hong IH (2008). Bone-protecting effect of *Rubus coreanus* by dual regulation of osteoblasts and osteoclasts. Menopause.

[6] Kim KJ, Jeong ES, Lee KH, Na JR, Park S, Kim JS (2020). Unripe *Rubus coreanus* miquel extract containing ellagic acid promotes lipolysis and thermogenesis in vitro and in vivo. Molecules.

[7] Kim Y, Lee SM, Kim JH (2014). Unripe *Rubus coreanus* miquel suppresses migration and invasion of human prostate cancer cells by reducing matrix metalloproteinase expression. Biosci. Biotechnol. Biochem..

[8] Ko SH, Chol SW, Ye SK, Yoo S, Kim HS, Chung MH (2008). Comparison of anti-oxidant activities of seventy herbs that have been used in Korean traditional medicine. Nutr. Res. Pract..

[9] Davel AP, Brum PC, Rossoni LV (2014). Isoproterenol induces vascular oxidative stress and endothelial dysfunction via a Gialphacoupled beta2-adrenoceptor signaling pathway. PLoS One.

[10] Srivastava S, Chandrasekar B, Gu Y, Luo J, Hamid T, Hill BG (2007). Downregulation of CuZn-superoxide dismutase contributes to beta-adrenergic receptor-mediated oxidative stress in the heart. Cardiovasc. Res..

[11] Rathore N, John S, Kale M, Bhatnagar D (1998). Lipid peroxidation and antioxidant enzymes in isoproterenol induced oxidative stress in rat tissues. Pharmacol. Res..

[12] Manjula TS, Devi CS (1993). Effect of aspirin on isoproterenol induced changes in lipid metabolism in rats. Indian J. Med. Res..

[13] Tayeb W, Nakbi A, Cheraief I, Miled A, Hammami M (2013). Alteration of lipid status and lipid metabolism, induction of oxidative stress and lipid peroxidation by 2,4-dichlorophenoxyacetic herbicide in rat liver. Toxicol. Mech. Methods.

[14] Pandey KB, Rizvi SI (2009). Plant polyphenols as dietary antioxidants in human health and disease. Oxid. Med. Cell. Longev..

[15] Kannan MM, Quine SD (2011). Ellagic acid ameliorates isoproterenol induced oxidative stress: evidence from electrocardiological, biochemical and histological study. Eur. J. Pharmacol..

[16] Yeager JC, Iams SG (1981). The hemodynamics of isoproterenol-induced cardiac failure in the rat. Circ. Shock.

[17] Upaganlawar A, Gandhi C, Balaraman R (2009). Effect of green tea and Vitamin E combination in isoproterenol induced myocardial infarction in rats. Plant Foods Hum. Nutr..

[18] Laine GA, Allen SJ (1991). Left ventricular myocardial edema. Lymph flow, interstitial fibrosis, and cardiac function. Circ. Res..

[19] Judd JT, Wexler BC (1974). Myocardial glycoprotein changes with isoproterenol-induced necrosis and repair in the rat. Am. J. Physiol..

[20] Sabeena Farvin KH, Anandan R, Kumar SH, Shiny KS, Sankar TV, Thankappan TK (2004). Effect of squalene on tissue defense system in isoproterenol-induced myocardial infarction in rats. Pharmacol. Res..

[21] Chae HJ, Yim JE, Kim KA, Chyun JH (2014). Hepatoprotective effects of *Rubus coreanus* miquel concentrates on liver injuries induced by carbon tetrachloride in rats. Nutr. Res. Pract..

[22] Nardi GM, Farias Januario AG, Freire CG, Megiolaro F, Schneider K, Perazzoli MR (2016). Anti-inflammatory activity of berry fruits in mice model of inflammation is based on oxidative stress modulation. Pharmacognosy Res..

[23] Forni C, Facchiano F, Bartoli M, Pieretti S, Facchiano A, D'Arcangelo D (2019). Beneficial role of phytochemicals on oxidative stress and age-related diseases. Biomed. Res. Int..

[24] Hasan R, Lasker S, Hasan A, Zerin F, Zamila M, Chowdhury FI (2020). Canagliflozin attenuates isoprenaline-induced cardiac oxidative stress by stimulating multiple antioxidant and anti-inflammatory signaling pathways. Sci. Rep..

[25] Marrocco I, Altieri F, Peluso I (2017). Measurement and clinical significance of biomarkers of oxidative stress in humans. Oxid. Med. Cell. Longev..

[26] Mohamadin AM, Elberry AA, Mariee AD, Morsy GM, Al-Abbasi FA (2012). Lycopene attenuates oxidative stress and heart lysosomal damage in isoproterenol induced cardiotoxicity in rats: a biochemical study. Pathophysiology.

[27] Chen W, Liang J, Fu Y, Jin Y, Yan R, Chi J (2020). Cardioprotection of cortistatin against isoproterenol-induced myocardial injury in rats. Ann. Transl. Med..

[28] Zhang GX, Kimura S, Nishiyama A, Shokoji T, Rahman M, Yao L (2005). Cardiac oxidative stress in acute and chronic isoproterenol-infused rats. Cardiovasc. Res..

[29] Blasig IE, Blasig R, Lowe H (1984). Myocardial lipid peroxidation during isoproterenol-induced blood flow reduction in rat myocardium. Biomed. Biochim. Acta.

[30] Zhou R, Xu Q, Zheng P, Yan L, Zheng J, Dai G (2008). Cardioprotective effect of fluvastatin on isoproterenol-induced myocardial infarction in rat. Eur. J. Pharmacol..

[31] Wang SB, Tian S, Yang F, Yang HG, Yang XY, Du GH (2009). Cardioprotective effect of salvianolic acid A on isoproterenol-induced myocardial infarction in rats. Eur. J. Pharmacol..

[32] Yogeeta SK, Hanumantra RB, Gnanapragasam A, Senthilkumar S, Subhashini R, Devaki T (2006). Attenuation of abnormalities in the lipid metabolism during experimental myocardial infarction induced by isoproterenol in rats: beneficial effect of ferulic acid and ascorbic acid. Basic Clin. Pharmacol. Toxicol..

[33] Rajadurai M, Stanely Mainzen Prince P (2006). Preventive effect of naringin on lipids, lipoproteins and lipid metabolic enzymes in isoproterenol-induced myocardial infarction in Wistar rats. J. Biochem. Mol. Toxicol..

[34] Tosheska Trajkovska K, Topuzovska S (2017). High-density lipoprotein metabolism and reverse cholesterol transport: strategies for raising HDL cholesterol. Anatol. J. Cardiol..

[35] Feng CC, Liao PH, Tsai HI, Cheng SM, Yang LY, PadmaViswanadha V (2018). Tumorous imaginal disc 1 (TID1) inhibits isoproterenol-induced cardiac hypertrophy and apoptosis by regulating c-terminus of hsc70-interacting protein (CHIP) mediated degradation of Galphas. Int. J. Med. Sci..

[36] Selvaraj P, Pugalendi KV (2012). Efficacy of hesperidin on plasma, heart and liver tissue lipids in rats subjected to isoproterenolinduced cardiotoxicity. Exp. Toxicol. Pathol..

[37] Devika PT, Stanely Mainzen Prince P (2008). (-)Epigallocatechin-gallate (EGCG) prevents mitochondrial damage in isoproterenolinduced cardiac toxicity in albino Wistar rats: a transmission electron microscopic and in vitro study. Pharmacol. Res..

[38] Garg S, Malhotra RK, Khan SI, Sarkar S, Susrutha PN, Singh V (2019). Fisetin attenuates isoproterenol-induced cardiac ischemic injury in vivo by suppressing RAGE/NF-kappaB mediated oxidative stress, apoptosis and inflammation. Phytomedicine.

[39] Teng H, Lin Q, Li K, Yuan B, Song H, Peng H (2017). Hepatoprotective effects of raspberry (*Rubus coreanus* miq.) seed oil and its major constituents. Food Chem. Toxicol..

[40] Yang JW, Choi IS (2016). Comparison of the phenolic composition and antioxidant activity of Korean black raspberry, Bokbunja, (*Rubus coreanus* miquel) with those of six other berries. CYTA - J. Food.

